# Increased Consumption of Sulfur Amino Acids by Both Sows and Piglets Enhances the Ability of the Progeny to Adverse Effects Induced by Lipopolysaccharide

**DOI:** 10.3390/ani9121048

**Published:** 2019-11-29

**Authors:** Ying Zhang, Bao-Yang Xu, Ling Zhao, Luo-Yi Zhu, Dolores Batonon-Alavo, Jeremy Jachacz, De-Sheng Qi, Shu-Jun Zhang, Li-Bao Ma, Lv-Hui Sun

**Affiliations:** 1 Department of Animal Nutrition and Feed Science, College of Animal Science and Technology, Huazhong Agricultural University, Wuhan 430070, China; zhangysunny@163.com (Y.Z.); baoyangxu@126.com (B.-Y.X.); lingzhao@webmail.hzau.edu.cn (L.Z.); zhuluoyi@outlook.com (L.-Y.Z.); qds@mail.hzau.edu.cn (D.-S.Q.); 2 Key Lab of Breeding and Reproduction of Ministry of Education, Huazhong Agricultural University, Wuhan 430070, China; sjxiaozhang@mail.hzau.edu.cn; 3 Adisseo France S.A.S., 10, Place du Général de Gaulle, 92160 Antony, France; dolores.batonon-alavo@adisseo.com (D.B.-A.); jeremy.jachacz@inra.fr (J.J.)

**Keywords:** sows, piglets, methionine, lipopolysaccharide, performance

## Abstract

**Simple Summary:**

Our results suggest that maternal consumption of total sulfur amino acids exceeding the NRC 2012 recommendations by 25% during late gestation and lactation benefits sow productivity and piglet neonatal performance. Moreover, increased consumption of sulfur amino acids by both sows and post-weaned piglets improved their ability to counteract the adverse effects by lipopolysaccharide (LPS) exposure. In addition, OH-Met showed a better response than DL-Met in both neonatal and weaned piglets. Taken together, our findings indicate that it might be necessary to update the recommendations for sulfur amino acids for gestating and lactating sows. Attention should also be given to sulfur amino acids supply during an inflammatory challenge as often encountered by piglets early in life.

**Abstract:**

This study determined the effects of increased consumption of sulfur amino acids (SAA), as either DL-Met or Hydroxy-Met (OH-Met), by sows and piglets on their performance and the ability of the progeny to resist a lipopolysaccharide (LPS) challenge. Thirty primiparous sows were fed a diet adequate in SAA (CON) or CON + 25% SAA, either as DL-Met or OH-Met from gestation day 85 to postnatal day 21. At 35 d old, 20 male piglets from each treatment were selected and divided into 2 groups (*n* = 10/treatment) for a 3 × 2 factorial design [diets (CON, DL-Met or OH-Met) and challenge (saline or LPS)]. OH-Met and/or DL-Met supplementation increased (*p* ≤ 0.05) piglets’ body weight gain during day 0–7 and day 7–14. Sow’s milk quality was improved in the supplemented treatments compared to the CON. The LPS challenge decreased (*p* ≤ 0.05) piglets’ performance from 35 to 63 d and increased (*p* ≤ 0.05) the levels of aspartate aminotransferase, total bilirubin, IL-1β, IL-6, TNF-a, and malondialdehyde. Plasma albumin, total protein, total antioxidant capacity and glutathione peroxidase decreased post-challenge. The results were better with OH-Met than DL-Met. The increase of Met consumption, particularly as OH-Met increased piglets’ growth performance during the lactation phase and the challenging period.

## 1. Introduction

Livestock husbandry practices have prompted an expanded use of synthetic amino acids in animal diets to enhance the performance and carcass quality of livestock, as well as to minimize the environmental impact caused by nitrogen excretion [1,2,3,4]. This is true for methionine, which is an essential amino acid for all livestock species, and it is the first limiting amino acid in diets for poultry and the second or third limiting amino acid in the corn-soybean meal diets for pigs [5,6]. Methionine is not only used for protein synthesis, but it is also involved in the methylation reactions of DNA [7,8] and in choline metabolism [9,10]. Methionine plays a role in antioxidant defense and acts as the precursor for bioactive compounds such as glutathione (GSH) and taurine [11,12]. 

Conventional sources of supplemental methionine used in animal feeds are either DL-methionine (DL-Met) or 2-hydroxy-4-methylthiobutanoic acid (OH-Met) [13]. Although these two compounds both provide L-methionine to various animal species, they are chemically different; OH-Met has a hydroxyl group at the asymmetric carbon atom, whereas DL-Met has an amino group. This chemical difference results in numerous differences with respect to the chemistry, absorption, transport in the body and metabolism by tissues [13]. Indeed, DL-Met is absorbed by active transport, whereas, OH-Met was absorbed by both active transport (Monocarboxylate transporter 1) and passive diffusion [14]. A previous study showed that increased consumption of methionine as OH-Met increased milk fat, lactose, cysteine, and taurine concentrations in OH-Met diet-fed sows [15]. The body weight of suckling piglets at two weeks of age in the OH-Met group was higher than that of the control group and tended to be higher than that of the DL-Met group [16]. However, there is a lack of studies for sows during late gestation regarding the efficacy of OH-Met as a source of methionine relative to DL-Met. 

In addition, weaning, when performed at very young age, is a period of physiological stress for piglets that predisposes them to enteric disease caused by pathogens such as *Escherichia coli* 0149 [17]. During *E. coli* infection, the bacterial endotoxin lipopolysaccharide (LPS) activates cells of the innate immune system leading to inflammation. Prolonged and/or excessive inflammation caused by stimulants such as LPS can adversely affect animal productivity by inducing muscle catabolism, anorexia and oxidative stress that is damaging to various organs [18]. According to previous reports, the demand for sulfur amino acids (SAAs), most notably cysteine, increases during immune responses [19,20]. Methionine is a precursor of cysteine and a potent antioxidant and immunity regulator [21,22]. Also, supplementing sow diets with extra methionine above the recommendations during gestation and lactation may provide additional methionine to piglets via the placenta and milk. Therefore, we hypothesized that supplementing methionine above the recommendations in the diets of sows during gestation and lactation could help their progeny counteract the adverse effects of oxidative stress and inflammation induced by LPS. In addition, with regards to the previous studies [15,16], we hypothesized that the better transulfuration obtained with OH-Met could lead to better results in comparison to DL-Met.

This study first aimed to compare the effects of an increase in dietary methionine as OH-Met or DL-Met on the performance of sows and their progeny during gestation and lactation. The second objective of this study was to assess the effects of LPS-induced inflammation and oxidative stress on piglets’ responses when they were fed increased levels of total SAAs, through either DL-Met or OH-Met diets, for a prolonged period of time during the post-weaning period.

## 2. Materials and Methods 

### 2.1. Animals, Treatments and Sample Collection

Our animal protocol was approved by the Institutional Animal Care and Use Committee of Huazhong Agricultural University, China (ethical approval code (if they do not have the code but you saved the file they sent to you, it is also fine to leave it like this)). In total, 30 primiparous sows (Landrace × Yorkshire) were randomly allocated to three treatment groups (*n* = 10 pens/treatment, 1 sow/pen) on gestation d 85 based on their body weight and backfat thickness (Supplemental Figure S1). Each sow was housed in a gestation crate from day 85 to 110 then in farrowing crate from day 110 of gestation to day 21 of lactation. Sows during gestation were allowed free access to water and mash feed; Sows were fed 1.0 kg feed at the first day of lactation, and feed was increased by 1.0 kg every day until day 7 of lactation. Free access to feed was given from 8 to 21 days of lactation. The control group of sows was fed a corn/soybean-control diet (CON) formulated to meet the nutritional requirements of sows (NRC, 2012, Table 1). The other two groups of sows were fed the control diet supplemented with either DL-Met (Rhodimet NP99 Adisseo, Antony, France) or OH-Met (Rhodimet AT88 Adisseo, Antony, France) at 25% above the total SAAs present in the control diet. The dose was chosen based on previous studies that reported that a dietary supplementation of an additional 25% total SAAs (TSAAs) during lactation improved the milk quality of sows and the antioxidant capacity of their progeny [15,16]. Within 12 h of farrowing, all litters were standardized to have 10 piglets per sow according to the average body weight of the piglets. Body weight and feed intake data of sows and their progeny were measured by a weighbridge during gestation and lactation. The backfat thickness of sows was measured 65 mm from the left side of the dorsal midline at the last rib level (P2) using an ultrasound (Lean-Meater, Renco, MN, USA) [16]. Blood was collected from the anterior vena cava of sows and piglets that were feed-deprived overnight for 8 h on gestation d 85 and lactation d 0 and 14. The plasma was prepared by centrifugation in heparinized tubes at 1000× *g* for 15 min at 4 ˚C and stored at −80 ˚C [22]. Colostrum was collected from each sow within 4 h of farrowing of the first piglet; milk was also collected from each sow after being feed-deprived overnight for 8 h on lactation day 14, as described previously [16]; the milk was stored at −80 ˚C before use.

On day 21, piglets were weaned, and piglets from the same sow were kept in a pen (Supplemental Figure S1). Piglets were not castrated. Piglets from the CON group of sows were fed antibiotic-free control diet (CON) formulated to meet their nutritional requirements (NRC, 2012, Table 1). Piglets from the DL-Met or OH-Met groups of sows were fed the CON supplemented with either DL-Met (DL-Met) or OH-Met (OH-Met) at 25% above the total SAAs present in the CON. The body weight of piglets was measured by a weighbridge at day 21 and 35. Piglets were allowed free access to the mash feed and water. Feed intake was measured for the 21–35 d period. 

On the morning of day 35, 60 male piglets from the three dietary treatments (20 piglets/treatment) were selected according to their body weight (Supplemental Figure S1). The initial body weights of the piglets were standardized and did not significantly differ among the 6 groups. They were divided into 6 groups (*n* = 10/group) for a 3 × 2 factorial design trial that included the dietary treatments (CON, DL-Met and OH-Met) and immunological challenge (saline vs. LPS (100 μg/kg BW, *E. coli* 0111: B4, Sigma)) by intraperitoneal injection. The dose of LPS was chosen in accordance with previous studies [23]. Blood was obtained from all piglets at 0 h pre-challenge and 4, 12, and 24 h post challenge to assess the acute phase response. Then, the piglets from the 6 groups (*n* = 5 pens/groups, 2 piglets/pen) were allowed free access to the same mash diets as before the immunological challenge for 4 weeks. The body weight and feed intake were measured biweekly from day 35 to day 63.

### 2.2. Milk Composition and Amino Acid Analysis 

The concentrations of fat, lactose, protein, and nonfat solid in milk were analyzed using an ultrasonic milk analyzer (MILKYWAY-CP2; Hangzhou Simple Technology Company, Limited, Hangzhou, China) as described previously [15]. The free amino acid concentration in milk was measured, as described previously [24]. Briefly, 1.0 mL milk was mixed with 1.0 mL n-hexane and centrifuged at 12,000× g for 5 min at 4 ˚C to remove fat. Then, 1.0 mL of the lower layer liquid was thoroughly mixed with 1.0 mL 5.0% sulfosalicylic acid and centrifuged at 12,000× g for 15 min at 4 ˚C to remove proteins. Finally, the supernatant was collected and filtered using a 0.22 μm millipore filter for free amino acid analysis using the Sykam S-433D automatic amino acid analyzer according to the ninhydrin postcolumn derivatization method.

### 2.3. Plasma Biochemical and Antioxidant Parameter Analysis

The concentrations of interleukin-1 beta (IL-1β), interleukin-6 (IL-6) and tumor necrosis factor-a (TNF-a) in plasma were measured using an ELISA kit with catalog numbers PLB00B, P6000B, and PTA00 from R&D Systems (USA). The concentrations of total protein (TP), albumin (ALB) and total bilirubin (TBIL) and activities of alanine aminotransferase (ALT) and aspartate aminotransferase (AST) were measured using a colorimetric method with specific assay kits (A045-2, A028-1, C019-1, C009-2 and C010-2) from the Nanjing Jiancheng Bioengineering Institute of China [25,26]. The activities of glutathione peroxidase (GPX) and total antioxidant capacity (T-AOC) and concentrations of GSH, glutathione disulfide (GSSG) and malondialdehyde (MDA) were measured using a colorimetric method with specific assay kits (A005, A015-1, A006-1, A061-2, and A003-1) from the Nanjing Jiancheng Bioengineering Institute of China [27].

### 2.4. Statistical Analyses of Results

Statistical analysis was performed using XLSTAT (Version 2015.3.01.19199). Growth performance data, backfat thickness and litter size generated from the sows and progeny during the lactation period were analyzed by one-way ANOVA using the Equation (1).

(1) $$Y_{ij}=\;\propto \;+\;\beta X_{i}+\varepsilon_{ij}$$

where $Y_{ij}$ = the response variable for treatment i, $X_{i}$ = treatment effect (i = CON, DL-Met, OH-Met), j being the experimental unit number, $\varepsilon_{ij}$ = residual of the model. 

An ANCOVA model was applied to body weight data generated during the LPS challenge, taking the treatments (CON, DL-Met, OH-Met) and the challenge (saline vs. LPS) as qualitative variables and the body weight at d 35 as a quantitative variable, according to Equation (2):

(2) $$Y_{ijk}=\;\propto \;+\;\beta X_{i}+\delta Z_{j}+BW_{k}+\theta XZ_{ij}+\varepsilon_{ijk}$$

where $Y_{ijk}$ = the response variable for treatment i, challenge j and the initial body weight k; $X_{i}$ = treatment effect (i = CON, DL-Met, OH-Met), $Z_{j}$ = challenge effect (j = LPS, Saline); $BW_{k}$ = the body weight for the kth individual at 35 days old; $XZ_{ij}$ is the interaction between the treatment and the challenge, $\varepsilon_{ijk}$ = residual of the model. 

The growth performance data and biochemistry data generated during the challenge were submitted to a two-way ANOVA using the following model described in Equation (3):

(3) $$Y_{ij}=\;\propto \;+\;\beta X_{i}+\delta Z_{j}+\theta XZ_{ij}+\varepsilon_{ij}$$

where $Y_{ijk}$ = the response variable for treatment *i*, challenge *j*; $X_{i}$ = treatment effect (i = CON, DL-Met, OH-Met), $Z_{j}$ = challenge effect (j = LPS, Saline; $XZ_{ij}$ is the interaction between the treatment and the challenge, $\varepsilon_{ij}$ = residual of the model.

*p* ≤ 0.05 was considered significant, and *p* ≤ 0.10 was considered to have a tendency toward difference. If there was a significant effect, a Tukey test was used for post hoc comparisons of means. Results were presented as means with SEM.

## 3. Results 

### 3.1. Performance of Sows and Piglets during the Late Gestation Phase, throughout the Lactation Phase and Day 21 to 35 

Table 2 presents the performance of the sows during the gestation and lactation periods. No significant differences (*p* > 0.05) were found in body weight and backfat thickness of the sows among the three groups at lactation d 0 and d 21. However, sow body weight loss between lactation d 0 and d 21 tended to be reduced with OH-Met (*p* = 0.08) compared to CON, whereas a significant effect of OH-Met was observed on the loss in back fat thickness (*p* ≤ 0.05). The average daily feed intake was not affected by dietary treatments from gestation d 85 to gestation d 114 and from d 0 to d 21 of lactation. Litter size at birth was not different among the treatments.

The piglets’ body weights at birth was not significantly different between the three treatments (Table 2). However, during lactation day 0–7, OH-Met increased the body weight gain of piglets (*p =* 0.10) by 62% in comparison to the CON. During lactation day 7–14, in comparison to the CON, DL-Met resulted in a 55% increase in piglet body weight gain, whereas OH-Met treatment resulted in a 63% increase. No significant effect of the treatments was observed on piglet mortality during the lactation period. At 35 d old, after two weeks of post-weaning feed supply, the piglets’ body weight, feed intake and feed conversion ratio were not significantly different between treatments. However, OH-Met tended (*p* = 0.09) to increase body weight in comparison to the CON (Table 2). 

#### Milk Composition and Free Amino Acid Concentrations 

Milk composition at lactation d 0 and d 14 is presented in Table 3. The supplemented treatments tended to increase milk protein (*p* = 0.07) and lactose (*p* = 0.09) concentrations on lactation d 0. Nonfat solid content was significantly increased (*p* ≤ 0.05) with both DL-Met and OH-Met, in comparison to the CON. On lactation day 14, milk fat content was similar between treatments. Milk protein concentration was significantly increase with OH-Met supplementation (5.64 ± 0.34%) in comparison to the CON (4.48 ± 0.23%), while DL-Met led to intermediate results (5.22 ± 0.52%). Lactose and nonfat solids were significantly increased with the increasing HO-methionine level but not with DL-Met in comparison to control.

Compared with the CON, DL-Met, and OH-Met enhanced (*p* ≤ 0.05) the milk free amino acid concentrations of cystine, glutamic acid, isoleucine, leucine, lysine, methionine, threonine, tyrosine, α-amino-n-butyric acid, β-amino-isobutyric acid, and taurine and decreased (*p* ≤ 0.05) ornithine and 3-methyl histidine concentrations at lactation d 0 and/or 14 (Table 4). Compared with the DL-Met group, the OH-Met group had greater (*p* ≤ 0.05) milk free amino acid concentrations of cystine, isoleucine, leucine, lysine, methionine, tyrosine, and α-amino-n-butyric acid but lower (*p* ≤ 0.05) ornithine and β-amino-isobutyric acid concentrations at lactation d 0 and/or 14 (Table 4). In contrast, the rest of the free amino acid concentrations in the milk of sows at the assayed time points were not affected by the dietary methionine supplementation (Supplementary Table S1).

### 3.2. Performance of Piglets after LPS Challenge from 35 to 63 Days Old

The growth performance of the piglets was significantly affected by the LPS challenge, diet, or their interaction (Table 5). The 6 groups had similar standardized initial body weight of piglets. Compared with the saline injection, the LPS challenge decreased body weight gain (*p* ≤ 0.05) and feed intake (*p* ≤ 0.05) and tended to decrease the gain-to-feed ratio (*p* ≤ 0.10) of piglets from day 35 to 49 and from day 49 to 63, respectively. Notably, the changes in the growth performance variables with the LPS challenge were attenuated (*p* ≤ 0.05) in piglets fed DL-Met and OH-Met compared to pigs fed the CON. Piglets fed increased levels of SAAs had higher body weight, body weight gain and gain-to-feed ratios than piglets fed the CON (*p* < 0.05) under the LPS challenge. The highest performance was obtained with OH-Met.

### 3.3. Plasma Biochemistry of Piglets after LPS Challenge 

The plasma biochemical variables of the piglets were significantly affected by the LPS challenge, diet, or their interaction (Table 6). Compared with the saline injection, the LPS challenge led to increased (*p* ≤ 0.05) activity of AST and concentration of TBIL and decreased (*p* ≤ 0.05) concentrations of ALB and TP in plasma at 4, 12, and/or 24 h post-LPS challenge. Meanwhile, the LPS challenge led to increased plasma levels of pro-inflammatory cytokines IL-1β, IL-6 and TNF-a at 4 h post-LPS challenge compared with the saline-treated group. Changes to most of these plasma biochemical variables due to the LPS challenge were alleviated in piglets fed DL-Met or OH-Met compared to pigs fed the CON. Piglets fed OH-Met exhibited similar values as the CON for some plasma biochemistry variables in comparison to piglets fed DL-Met at various time points after the LPS challenge. 

### 3.4. Plasma Antioxidant Parameters of Piglets After LPS Challenge 

The plasma antioxidant variables of the piglets were significantly affected by the LPS challenge or diet (Table 7). Compared with the saline injection among pigs fed the CON, the LPS challenge led to decreased (*p* ≤ 0.05) activities of T-AOC (27.0–36.0%) and GPX (~15.7%), and an increased (*p* ≤ 0.05) MDA (36.8–57.9%) concentration in plasma at 12 and/or 24 h post-LPS challenge. Notably, changes to plasma T-AOC and GPX activities due to the LPS challenge were prevented (*p* < 0.05), but the MDA concentration was not alleviated in piglets fed DL-Met and OH-Met. 

## 4. Discussion

The present study shows that maternal and neonatal methionine supplementation, particularly in the form of OH-Met, during late gestation, lactation, and postweaning, can improve the performance of sows and their progeny. Dietary supplementation of both DL-Met and OH-Met did not affect body weight, backfat thickness, feed intake and litter size at birth for the sows, but OH-Met supplementation reduced the loss of body weight and backfat thickness of sows during lactation. A previous study also showed that dietary supplementation of DL-Met and OH-Met during lactation did not affect the body weight and backfat thickness of sows, but the loss of body weight and backfat during lactation was not calculated [15]. Because loss of body weight and backfat during lactation reduces subsequent reproductive performance [28,29], the present study offers the first evidence that dietary supplementation of OH-Met during later gestation and lactation benefits body energy metabolism and can potentially improve the reproduction of sows. Likewise, dietary supplementation of DL-Met and(or) OH-Met increased the body weight gain of piglets during day 0–7 and day 7–14. These outcomes were similar to a previous study, in which dietary supplementation of OH-Met during lactation increased the body weight of piglets at day 14, while DL-Met supplementation did not affect piglet body weight [15]. This discrepancy could be attributed to the different dietary methionine supplementation period. Indeed, dietary supplementation of both DL-Met and OH-Met increased milk protein, lactose, and/or nonfat solid at lactation day 0, but only OH-Met supplementation increased milk protein, lactose, and nonfat solids at lactation d 14; these results are similar to those of previous studies performed in cows and sows [15,30,31]. Supplementation of methionine increased milk quality and may be associated with the mechanisms: (1) methionine supplementation increased the level of arterial concentrations of methionine, which improved the amino acid supply to the mammary gland for milk synthesis [31]; (2) the HMTBA potential increase the blood flow may also contribute to increased milk production [15]; (3) increasing milk lactose and protein levels positively correlates with milk yield [32]. These data indicate that maternal dietary methionine supplementation might improve the body weights of piglets. 

Thirteen free amino acids present in milk were affected by maternal dietary methionine supplementation. Among them, five essential amino acids (isoleucine, leucine, lysine, methionine, threonine) and a nonessential amino acid (tyrosine) are used by cells to synthesize proteins and play important roles in the activation of protein biosynthesis [33]. Thus, increasing these amino acids in the milk by maternal dietary DL-Met or OH-Met supplementation could contribute to increasing the body weight gain of piglets. The increased concentrations of these amino acids were associated with a decrease of glutamic acid in milk in the DL-Met and OH-Met groups. The lower milk concentration of glutamic acid in the DL-Met and OH-Met groups was the result of the lower levels of glutamic acid in sows’ plasma (data not shown). Glutamic acid is known to be the major fuel of the gut, which means that a higher proportion of glutamic acid was metabolized by the intestine in the first-pass [34]. Therefore, the increase in glutamic acid catabolism might have reduced the catabolism of dietary-essential amino acids as energy substrates for the intestinal mucosa, thus explaining the increased concentrations of amino acids in sows’ plasma and milk, as shown in multicatheterized piglets [34]. However, lower milk glutamic acid levels at lactation day 14 in the DL-Met and OH-Met groups might also compromise the benefits of the improved milk quality on growth performance for piglets. Indeed, the body weight gain of piglets during day 14–21 did not increase in the DL-Met and OH-Met groups in the current study. Nevertheless, the mechanism of Met increases glutamic acid catabolism in the gut and needs to be further explored. In addition, 3-methyl histidine, a muscular proteolysis marker [35], was reduced in the milk of sows in the DL-Met and OH-Met groups, which indicated less muscle wasting in sows of the DL-Met and OH-Met groups relative to those of the CON group. These results might potentially explain the lower losses of body weight and backfat thickness of sows during lactation in the dietary OH-Met supplementation group. However, since dietary DL-Met supplementation had the same effects on milk glutamic acid and 3-methyl histidine as OH-Met without significantly affecting body weight and backfat thickness of sows during lactation, this finding would need to be further explored in additional studies.

Maternal dietary DL-Met and/or OH-Met supplementation also increased free amino acids in milk that were associated with redox control (cystine and taurine), which might enhance the ability of piglets to cope with stress [36]. Additionally, maternal dietary methionine supplementation increased free amino acids in milk that play roles in the nonribosomal peptide synthases (α-amino-n-butyric acid) and cell metabolism (β-amino-isobutyric acid) and decreased free amino acids in milk that play roles in the urea cycle (ornithine) [1,37]. The actual contribution of these alterations to amino acids in milk on the performance of sows and piglets still need to be further studied.

Another novel finding in the current study was that methionine, particularly in the form of OH-Met, alleviated LPS-induced adverse effects in piglets. The LPS challenge reduced body weight gain, feed intake and feed utilization efficiency throughout the study in piglets, in accordance with earlier studies [38]. Strikingly, changes to these growth performance variables due to the LPS challenge were alleviated by dietary OH-Met supplementation, while only the changes to the gain-to-feed ratio during d 49–63 due to the LPS challenge were mitigated by dietary DL-Met supplementation. Moreover, the LPS challenge induced hepatic injury and inflammatory reactions that included increased AST activity and TBIL concentration and decreased ALB and TP concentrations in plasma at 4, 12 and/or 24 h post-LPS challenge; the plasma levels of pro-inflammatory cytokines IL-1β, IL-6 and TNF-a were also increased at 4 h post-LPS challenge. These outcomes were in accordance with previous studies [39,40,41,42]. Interestingly, methionine supplementation attenuated the plasma biochemistry changes induced by the LPS challenge, and piglets fed OH-Met displayed a stronger ability to mitigate these plasma biochemistry changes relative to piglets fed DL-Met. Furthermore, consistent with previous studies [18,43], the piglets challenged by LPS in this experiment experienced oxidative stress, as indicated by the reduction of antioxidant capacity (GPX and T-AOC) and increased lipid peroxidation (MDA), whereas dietary supplementation of both DL-Met and OH-Met prevented these changes in GPX and T-AOC. Meanwhile, piglets in the DL-Met and OH-Met groups fed milk and diets with higher cystine, taurine and methionine experienced enhanced antioxidant capacity [15,16]. Taken together, these outcomes agree with previous studies, which reported that LPS challenges reduce piglets’ growth performance in association with inflammation and oxidative stress. However, dietary methionine supplementation, especially in the form of OH-Met, showed protective actions against LPS-impaired growth performance in piglets, which was associated with an enhancement of immune function and antioxidant capacities.

## 5. Conclusions

Maternal and neonatal methionine supplementation during late gestation, lactation and postweaning improved the performance of sows and their progeny. This was mainly associated with improved milk quality, along with increased essential (isoleucine, leucine, lysine, methionine, threonine) and reduction-oxidative control (cystine and taurine) amino acids and decreased energy (glutamic acid) and muscular proteolysis markers (3-methyl histidine) amino acids in milk. Moreover, prolonged supplementation of methionine in the postweaning diet of the progeny improved their ability to counteract LPS-induced negative effects, which may be due to the enhancement of anti-inflammation and antioxidant capacities of piglets. Finally, better results were obtained with dietary supplementation of OH-Met in comparison to DL-Met for most of these variables, which indicates that OH-Met might be a better methionine source than DL-Met for pig production. This novel finding indicated that the current methionine requirement from NRC (2012) may be underestimated and could have meaningful impacts on swine nutrition and health.

## Figures and Tables

**Table 1:** Ingredients and nutrients composition of the Control diet offered to both sows and piglets ^1^.

Ingredients (%)	Sows		Piglets	
	Gestation	Lactation	Post-Weaning I (21–35 d Old)	Post-Weaning II (35–63 d Old)
Corn	61.77	65.74	17.60	60.70
Expanded corn	-	-	15.0	-
Wheat flour	-	-	10.0	-
Wheat bran	15	-	-	-
Soybean meal	14	28	-	27.5
Expanded soybeans	-	-	8.0	-
Fermented soybean meal	-	-	5.0	-
Corn gluten feed	2.0	-	-	-
Fish meal	-	-	4.0	5.0
Whey powder	-	-	12.0	-
Soybean oil	3.5	2.5	-	2.5
Sugar	-	-	8.0	-
Glucose	-	-	6.0	-
Emulsified fat powder	-	-	5.0	-
Plasma protein	-	-	5.0	-
CaCO_3_	1.00	0.60	0.50	0.50
CaHPO_4_	1.20	1.70	1.50	1.50
Salt	0.30	0.30	0.30	0.30
DL-Met	0.07	0.06	0.30	0.25
L-Lys	0.16	0.10	0.50	0.55
L-Thr	-	-	0.30	0.20
Vitamin premix ^2^	0.50	0.50	0.50	0.50
Mineral premix ^3^	0.50	0.50	0.50	0.50
Crude protein (%)	14.6	17.6	21.0	20.9
Digestible energy (MJ/kg)	13.7	14.2	14.2	14.0
Total Lys (%)	0.75	0.98	1.45	1.56
Total Met (%)	0.29	0.32	0.48	0.58
Total Met + Cys (%)	0.52	0.60	0.82	0.88
D Lys (%)	0.65	0.85	1.30	1.40
SID Met (%)	0.26	0.28	0.45	0.53
SID Met+Cys (%)	0.45	0.52	0.72	0.78
Calcium (%)	0.69	0.69	0.65	0.81
Total phosphorus (%)	0.60	0.63	0.64	0.73

^1^ The DL-Met and OH-Met treatment diets during gestation, lactation, and day 21–35 and day 35–63 were prepared by adding 1.313, 1.515, 2.071, or 2.22 kg DL-Met (99%) and 1.477, 1.705, 2.330, or 2.5 kg OH-Met (88%), respectively, to 1000 kg of the control diet at the expense of corn. These methionine sources addition leading to obtain TSSA levels in DL-Met and OH-Met treatments for gestation, lactation, and day 21–35 and day 35–63 are 0.65%; 0.65%; 1.04%; 1.00%, respectively. ^2^ Vitamin premix provided per kg of diet: retinyl acetate, 10000 IU; cholecalciferol 2500 IU; dl-α-tocopheryl acetate, 50 IU; menadione, 5.0 mg; thiamin, 2.0 mg; riboflavin, 5.0 mg; pantothenic acid, 12.0 mg; pyridoxine, 10.0 mg; niacin, 30.0 mg; *d*-biotin, 0.2 mg; folic acid, 1.5 mg; cyanocobalamin, 0.05 mg; choline chloride 1500 mg. ^3^ Mineral premix provided per kg of diet: FeSO_4_•7H_2_O, 498 mg; CuSO_4_•5H_2_O, 78.7 mg; MnSO_4_•5H_2_O, 110 mg; ZnSO_4_•7H_2_O, 440 mg; Na_2_SeO_3_, 0.66mg; KI, 0.4 mg.

**Table 2:** Performance of sows fed with diets supplemented with either DL-Methionine (DL-Met) or DL-2-hydroxy-4-methylthiobutanoic acid (OH-Met) at the requirements in TSAAs or above during the late gestation and lactation periods and their progeny ^1^.

Item	CON	DL-Met	OH-Met	SEM	*p*-Value
Sows (No. of sows)	10	10	10		
Body weight, kg					
Gestation day 85	174	172	170	4	0.720
Lactation day 0	179	174	171	4	0.337
Lactation day 21	173	170	171	5	0.879
Changes (Lactation day 0–21)	−6.29 *	−4.25	−0.33 *	0.24	0.235
Backfat thickness, mm					
Gestation day 85	17.4	18.3	17.7	0.9	0.800
Lactation day 0	18.5	19.5	19.0	1.0	0.766
Lactation day 21	16.5	17.9	18.4	0.9	0.349
Changes (Lactation day 0–21)	−2.00 ^†^	−1.61	−0.61 ^†^	0.41	0.068
Average daily feed intake, kg					
Gestation day 85–114	3.12	3.17	2.97	0.18	0.731
Lactation day 0–21	5.26	5.32	5.26	0.11	0.913
Litter size at birth					
Born alive, n	11.5	11.1	10.6	0.8	0.374
Stillborn,%	6.40	1.91	3.87	1.47	0.317
Mummies,%	0.00	0.00	0.83	0.28	0.381
Piglet’s body weight, kg					
Body weight at day 0	1.33	1.19	1.30	0.06	0.296
Body weight gain day 0–7, kg	0.68 ^#^	0.82	1.01 ^#^	0.08	0.207
Body weight gain day 7–14, kg	1.03 ^a^	1.60 ^b^	1.68 ^b^	0.09	0.009
Body weight gain day 14–21, kg	1.89	1.66	1.66	0.09	0.961
Piglet’s Mortality day 0–21, n	1.65	1.01	2.47	0.88	0.530
Post-weaning phase (day 21–35) ^2^					
Body weight day 35, kg	7.66 ^+^	8.25	8.47 ^+^	0.30	0.175
Body weight gain, kg	2.76	2.82	2.87	0.08	0.861
Feed intake day 35, kg	3.35	3.36	3.40	0.14	0.956
Feed conversion ratio day 21–35, kg/kg	1.22	1.20	1.19	0.04	0.841

^1^ Values are means ± SE, *n* = 10. Labeled means in a row with unlike superscript letters were significantly different by Tukey test (*p* < 0.05). *^, †, #, +^ Different by Tukey test: * *p* = 0.10; ^†^ *p* =0.03; ^#^ *p* = 0.10; ^+^ *p* = 0.09. CON = control diet; DL-Met = CON supplemented with DL-Met at 25% above the total sulphur amino acids present in the control diet; OH-Met = CON supplemented with OH-Met at 25% above the total sulphur amino acids present in the control diet. ^2^ Piglets were weaned at 21 d. They were fed with post-weaning diets according to their maternal feeding until day 35.

**Table 3:** Milk composition of sows at lactation day 0 and 14, when fed with diets supplemented with either DL-Methionine (DL-Met) or DL-2-hydroxy-4-methylthiobutanoic acid (OH-Met) at the requirements in TSAAs or above during the late gestation and lactation periods ^1^.

Item	CON	DL-Met	OH-Met	SEM	*p*-Value
Lactation day 0					
Fat, %	7.51	8.26	8.01	0.51	0.560
Protein, %	6.32 *^,†^	7.44 ^†^	7.92 *	0.48	0.074
Lactose, %	9.09 ^#^	10.2	11.3 ^#^	0.7	0.091
Nonfat solid, %	16.9 ^a,+^	19.8 ^b,+^	21.1 ^b^	1.2	0.049
Lactation day 14					
Fat, %	5.06	5.58	5.61	0.36	0.477
Protein, %	4.48 ^a^	5.22 ^ab^	5.64 ^b^	0.32	0.021
Lactose, %	5.68 ^a^	5.91 ^a^	7.36 ^b^	0.30	<0.01
Nonfat solid, %	10.7 ^a^	11.7 ^a^	14.7 ^b^	0.6	<0.001

^1^ Values are means ± SE, *n* = 10. ^a-b^ Labeled means in a row with unlike superscript letters were significantly different by Tukey test (*p* < 0.05). *^,†,#,+^ Different: * *p* = 0.03; ^†^ *p* = 0.09; ^#^ *p* = 0.03; ^+^ *p* = 0.10. CON = control diet; DL-Met = CON supplemented with DL-Met at 25% above the total sulphur amino acids present in the control diet; OH-Met = CON supplemented with OH-Met at 25% above the total sulphur amino acids present in the control diet.

**Table 4:** Effects of methionine supplementation on milk free amino acid concentrations of sows at lactation day 0 and 14 ^1^.

Compound, µmol/L	Lactation Day 0					Lactation Day 14				
	CON	DL-Met	OH-Met	SEM	*p* Value	CON	DL-Met	OH-Met	SEM	*p*-Value
Cystine	8.8 ^c^	13.4 ^b^	22.7 ^a^	2.0	<0.001	12.4	15.2	14.8	1.5	0.386
Glutamic acid	331	326	312	14	0.655	322 ^a^	235 ^b^	232 ^b^	14	<0.001
Isoleucine	14.1 ^c^	19.4 ^b^	29.6 ^a^	1.5	< 0.001	11.6 ^b^	19.3 ^a^	22.5 ^a^	2.0	0.002
Leucine	24.4	23.6	25.7	1.0	0.337	8.5 ^c^	18.3 ^b^	29.2 ^a^	1.2	<0.001
Lysine	235 ^c^	412 ^b^	525 ^a^	25	<0.001	36.7 ^c^	69.8 ^b^	104 ^a^	8.7	<0.001
Methionine	9.8 ^c^	36.0 ^b^	54.3 ^a^	2.1	<0.001	23.6	21.8	20.7	2.3	0.662
Ornithine	43.7 ^a^	31.4 ^b^	25.3 ^c^	1.7	<0.001	15.8 ^a^	13.7 ^a^	9.3 ^b^	1.5	0.016
Taurine	1465 ^b^	2157 ^a^	2023 ^a^	51	<0.001	857 ^b^	1309 ^a^	1294 ^a^	72	<0.001
Threonine	27.7 ^b^	39.7 ^a^	41.6 ^a^	1.7	<0.001	11.0	11.0	12.5	1.2	0.601
Tyrosine	15.8	16.3	16.4	0.7	0.746	14.5 ^b^	18.3 ^b^	25.9 ^a^	2.2	0.003
α-amino-n-butyric acid	2.6 ^c^	4.0 ^b^	5.0 ^a^	0.4	<0.001	2.1 ^c^	2.9 ^b^	3.7 ^a^	0.2	<0.001
β-amino-isobutyric acid	18.0 ^c^	57.0 ^a^	43.6 ^b^	3.3	<0.001	18.2	20.2	21.0	2.5	0.722
3-methyl histidine	1.8 ^a^	0.2 ^b^	0.3 ^b^	0.2	<0.001	1.2 ^a^	0.7 ^b^	0.5 ^b^	0.1	<0.001

^1^ Values are means ± SE, *n* = 10. ^a–b^ Labeled means in a row with unlike superscript letters were significantly different by Tukey test (*p* < 0.05). CON = control diet; DL-Met = CON supplement with DL-Met at 25% above the total sulphur amino acids present in the control diet; OH-Met = CON supplement with OH-Met at 25% above the total sulphur amino acids present in the control diet.

**Table 5:** Effects of methionine supplementation on performance of progeny after LPS challenge from 35 to 63 days old ^1^.

Item	Saline			LPS			SEM	*p*-Value		
	CON	DL-Met	OH-Met	CON	DL-Met	OH-Met		LPS	Diet	LPS × Diet
Body weight at day 35, kg	8.18	8.13	8.25	8.20	8.21	8.26	0.08	0.827	0.906	0.983
Day 35–49										
Body weight at day 49, kg	11.8 ^cd^	11.9 ^cd^	12.1 ^d^	10.3 ^a^	10.8 ^ab^	11.1 ^bc^	0.1	<0.001	0.053	0.400
Daily weight gain, kg	0.258 ^bc^	0.267 ^c^	0.274 ^c^	0.149 ^a^	0.182 ^a^	0.202 ^ab^	0.013	<0.001	0.040	0.369
Daily feed intake, kg	0.398 ^bc^	0.413 ^bc^	0.429 ^c^	0.267 ^a^	0.309 ^a^	0.338 ^ab^	0.019	<0.001	0.047	0.577
Gain:feed	0.645 ^b^	0.650 ^b^	0.638 ^b^	0.552 ^a^	0.590 ^ab^	0.598 ^ab^	0.017	<0.001	0.379	0.303
Day 49–63										
Body weight at day 49, kg	18.4 ^c^	18.6 ^c^	18.8 ^c^	14.7 ^a^	15.7 ^ab^	16.1 ^b^	0.3	<0.001	0.042	0.224
Daily weight gain, kg	0.474 ^b^	0.483 ^b^	0.476 ^b^	0.316 ^a^	0.349 ^a^	0.360 ^a^	0.020	<0.001	0.460	0.594
Daily feed intake, kg	0.843 ^b^	0.855 ^b^	0.834 ^b^	0.715 ^a^	0.676 ^a^	0.683 ^ab^	0.034	<0.001	0.827	0.751
Gain:feed	0.561 ^b^	0.565 ^b^	0.574 ^b^	0.444 ^a^	0.516 ^b^	0.527 ^b^	0.016	<0.001	0.014	0.058
Day 35–63										
Daily weight gain, kg	0.366 ^c^	0.375 ^c^	0.375 ^c^	0.233 ^a^	0.265 ^ab^	0.281 ^b^	0.011	<0.001	0.037	0.216
Daily feed intake, kg	0.621 ^b^	0.631 ^b^	0.634 ^b^	0.491 ^a^	0.493 ^a^	0.510 ^a^	0.015	<0.001	0.625	0.797
Gain:feed	0.589 ^bc^	0.590 ^bc^	0.595 ^bc^	0.474 ^a^	0.539 ^b^	0.550 ^bc^	0.012	<0.001	0.007	0.018

^1^ Values are means ± SE, *n* = 5. ^a-b^ Labeled means in a row with unlike superscript letters were significantly different (*p* < 0.05). CON = control diet; DL-Met = CON supplement with DL-Met at 25% above the total sulphur amino acids present in the control diet; LPS = lipopolysaccharide; OH-Met = CON supplement with OH-Met at 25% above the total sulphur amino acids present in the control diet.

**Table 6:** Effect of methionine supplementation on plasma biochemistry of progeny after LPS challenge at 35 d old ^1^.

Item	Saline			LPS			SEM	*p*-Value		
	CON	DL-Met	OH-Met	CON	DL-Met	OH-Met		LPS	Diet	LPS × Diet
ALT, U/L	52.5	53	48.3	49.4	49.5	52.5	4.1	0.816	0.979	0.583
AST, U/L	80.3	73.5	72.6	79.2	78.5	71.2	5.3	0.850	0.350	0.793
TBIL, umol/L	3.5	3.5	3.3	3.7	3.6	3.4	0.3	0.54	0.751	0.991
TP, g/L	51.9	49.8	48.7	52.6	49.4	52.8	2.6	0.492	0.592	0.655
ALB, g/L	27.5	27.8	27.2	29.4	28.2	28	1.4	0.383	0.859	0.860
IL-1β, ng/L	63	68.6	65.5	66.2	63.9	59.8	6.7	0.666	0.869	0.770
IL-6, ng/L	137	130	150	132	126	139	19	0.664	0.695	0.978
TNF-a, ng/L	290	254	287	280	257	262	27	0.649	0.552	0.882
4 h										
ALT, U/L	51.5	48.1	47.1	53.5	49.3	50.8	4.5	0.543	0.647	0.959
AST, U/L	79.6	80.4	77.6	87.4	84	80.2	6.0	0.352	0.738	0.902
TBIL, umol/L	3.5 ^a^	3.3 ^a^	3.2 ^a^	16.5 ^c^	12.1 ^bc^	10.0 ^b^	1.4	<0.001	0.063	0.088
TP, g/L	47.9	47.1	44.9	46.2	45	43.2	2.0	0.269	0.358	0.994
ALB, g/L	27.9 ^ab^	28.6 ^b^	26.6 ^ab^	22.7 ^a^	24.5 ^ab^	22.9 ^a^	1.2	<0.001	0.319	0.823
IL-1β, ng/L	64.5 ^a^	61.2 ^a^	59.9 ^a^	160 ^c^	136 ^bc^	125 ^b^	8	<0.001	0.048	0.157
IL-6, ng/L	124 ^a^	128 ^a^	120 ^a^	357 ^c^	302 ^bc^	255 ^b^	17	<0.001	0.018	0.028
TNF-a, ng/L	268 ^a^	291 ^a^	287 ^a^	2416 ^c^	1869 ^b^	1752 ^b^	115	<0.001	0.02	0.013
12 h										
ALT, U/L	43.6	44.1	46.8	43.4	47.9	41.3	5.5	0.884	0.893	0.701
AST, U/L	89.8 ^ab^	83.7 ^a^	80.2 ^a^	121 ^b^	104 ^ab^	96.7 ^ab^	8.5	<0.001	0.142	0.671
TBIL, umol/L	3.4 ^ab^	3.5 ^ab^	2.8 ^a^	14.3 ^d^	10.3 ^cd^	8.7 ^bc^	1.3	<0.001	0.067	0.142
TP, g/L	55.4 ^b^	53.9 ^b^	55.1 ^a^	45.3 ^a^	49.4 ^ab^	51.5 ^ab^	2.4	0.005	0.493	0.363
ALB, g/L	27.6	28.4	26.2	22.7	27.2	24.8	1.6	0.065	0.197	0.426
IL-1β, ng/L	57.8	59.4	64.6	66.5	65.7	58.7	6.6	0.573	0.99	0.499
IL-6, ng/L	120	106	119	128	117	114	11	0.597	0.492	0.723
TNF-a, ng/L	277	254	258	302	290	286	28	0.200	0.771	0.983
24 h										
ALT, U/L	52.2	44.8	45.7	46.9	48	47	5.5	0.798	0.956	0.718
AST, U/L	90.1 ^ab^	93.9 ^ab^	85.7 ^ab^	174 ^c^	148 ^bc^	80.9 ^a^	15.3	0.001	0.009	0.022
TBIL, umol/L	3.3 ^a^	3.6 ^a^	3.2 ^a^	8.9 ^b^	7.5 ^b^	5.7 ^ab^	0.8	<0.001	0.129	0.145
TP, g/L	46.4	45	48.9	41.1	45.1	47.5	2.5	0.286	0.196	0.535
ALB, g/L	25.4	27	24.1	26	26.3	27.3	2.5	0.632	0.918	0.747
IL-1β, ng/L	64.1	62.3	61.7	63.8	74.4	67.6	6.0	0.237	0.739	0.593
IL-6, ng/L	116	119	128	123	110	113	10	0.492	0.813	0.519
TNF-a, ng/L	247	257	249	264	262	235	28	0.094	0.995	0.885

^1^ Values are means ± SE, *n* = 5. ^a-b^ Labeled means in a row with unlike superscript letters were significantly different (*p* < 0.05). ALT = alanine aminotransferase; ALB = albumin; AST = aspartate aminotransferase; CON = control diet; DL-Met = CON supplement with DL-Met at 25% above the total sulphur amino acids present in the control diet; IL-1β = interleukin-1 beta; IL-6 = interleukin-6; LPS = lipopolysaccharide; OH-Met = CON supplement with OH-Met at 25% above the total sulphur amino acids present in the control diet; TBIL = total bilirubin; TP = total protein; TNF-a = tumor necrosis factor-a.

**Table 7:** Effect of methionine supplementation on plasma antioxidant parameters of progeny after LPS challenge at 35 d old ^1^.

Item	Saline			LPS			SEM	*p* Value		
	CON	DL-Met	OH-Met	CON	DL-Met	OH-Met		LPS	Diet	LPS x Diet
0 h										
GPX, U/mL	591	622	585	594	619	576	30	0.904	0.394	0.979
T-AOC, U/L	151	147	155	143	164	156	15	0.771	0.771	0.664
GSH, μmol/L	23.9	27.8	27.4	24.6	26.8	26.3	2.1	0.796	0.300	0.895
GSSG, μmol/L	2.3	2.8	2.5	3.0	2.6	2.3	0.4	0.773	0.742	0.478
MDA, nmol/L	1.6	1.5	1.6	1.7	1.6	1.9	0.2	0.321	0.490	0.877
4 h										
GPX, U/mL	615	636	612	607	670	600	29	0.819	0.211	0.665
T-AOC, U/L	164	158	172	156	146	157	15	0.337	0.701	0.974
GSH, μmol/L	24.6	21.5	23.4	22.9	20.9	24.2	2.1	0.768	0.373	0.829
GSSG, μmol/L	1.3	1.4	1.1	1.5	1.2	1.2	0.3	0.891	0.698	0.782
MDA, nmol/L	1.8	1.8	2	1.6	1.9	1.9	0.2	0.748	0.523	0.838
12 h										
GPX, U/mL	499	505	510	471	466	482	24	0.114	0.867	0.963
T-AOC, U/L	145 ^a^	134 ^a^	130 ^a^	92.8 ^b^	113 ^ab^	130 ^a^	13	0.03	0.695	0.140
GSH, μmol/L	13.3	15.0	12.9	12.0	14.8	13.0	2.1	0.785	0.515	0.934
GSSG, μmol/L	2.5	2.7	2.4	2.6	2.6	2.3	0.4	0.782	0.803	1.000
MDA, nmol/L	1.8 ^a^	2.2 ^ab^	1.8 ^b^	2.7 ^ab^	2.9 ^a^	2.6 ^ab^	0.2	< 0.001	0.234	0.914
24 h										
GPX, U/mL	458 ^a^	473 ^a^	467 ^a^	386 ^b^	421 ^ab^	426 ^ab^	24	0.008	0.479	0.803
T-AOC, U/L	124 ^a^	120 ^a^	131 ^a^	90.5 ^b^	108 ^ab^	109 ^ab^	13	0.04	0.627	0.700
GSH, μmol/L	10	10.8	9.8	7.1	9.5	10.3	1.5	0.334	0.493	0.547
GSSG, μmol/L	2.5	2.0	1.9	2.3	2.0	2.1	0.3	0.826	0.370	0.787
MDA, nmol/L	2.0 ^b^	1.9 ^b^	1.9 ^b^	2.9 ^a^	3.0 ^a^	2.6 ^a^	0.3	<0.001	0.771	0.820

^1^ Values are means ± SE, *n* = 5. ^a-b^ Labeled means in a row with unlike superscript letters were significantly different (*p* < 0.05). DL-Met = CON supplement with DL-Met at 25% above the total sulphur amino acids present in the control diet; GPX = glutathione peroxidase; GSH = glutathione; GSSG = glutathione disulfide; LPS = lipopolysaccharide; MDA = malondialdehyde; OH-Met = CON supplement with OH-Met at 25% above the total sulphur amino acids present in the control diet; T-AOC = total antioxidant capacity.

## Supplementary Materials

The following are available online at https://www.mdpi.com/2076-2615/9/12/1048/s1.
