# Supplementary material for: Increased Consumption of Sulfur Amino Acids by Both Sows and Piglets Enhances the Ability of the Progeny to Adverse Effects Induced by Lipopolysaccharide
Source: Animals (Basel). 2019 Nov 29;9(12):1048. doi: 10.3390/ani9121048 (PMC6940865; doi:10.3390/ani9121048)

Supplementary Table S1. Effects of methionine supplementation on milk free amino acid concentrations of sows at lactation d 0 and 14[1].

	Lactation d 0					Lactation d 14				
**Compound, μmol/L**	**CON**	**DL-Met**	**OH-Met**	**SEM**	**P-value**	**CON**	**DL-Met**	**OH-Met**	**SEM**	**P-value**
Alanine	112	106	105	4	0.449	172	207	187	20	0.442
Arginine	38.6	35.8	37.2	1.9	0.601	50	49.2	43.3	6.8	0.748
Asparagine	54.5	54.9	56.7	2.9	0.848	33.9	39	35.2	4.8	0.739
Aspartic Acid	249	230	231	11	0.393	23.3	27.1	22.7	2.9	0.506
Citrulline	20.2	21.2	21.3	1.1	0.735	6.2	7.2	6.6	0.5	0.274
Glutamine	147	160	155	8	0.513	185	178	186	14	0.891
Glycine	192	198	198	10	0.859	217	213	198	15	0.640
Histidine	15.9	15.9	14.7	0.9	0.528	19	23	20.9	2.1	0.433
Hydroxyproline	22.2	27.1	26.9	1.0	0.975	25.7	24.5	24.3	0.7	0.334
Phenylalanine	26.2	27.3	30	2.1	0.443	18.4	21.2	18.6	2.6	0.699
Phosphoserine	33.8	35.2	37.5	1.7	0.319	64	61.3	63.6	4.6	0.907
Phosphoethanolamine	615	591	644	25	0.329	678	646	581	49	0.377
Proline	58.7	57.8	56.1	3.3	0.857	54.6	53.8	53.4	3.9	0.975
Serine	39.1	40.2	43.2	2.3	0.419	55.4	50	57	5.9	0.688
Tryptophan	23.8	23.9	25.7	0.9	0.262	27.2	25.5	25.3	1.8	0.705
Valine	17	17.9	16.4	0.7	0.281	24	27.6	30.4	3.2	0.372
α-amino-adipic acid	10.1	9.7	10.3	0.6	0.802	5.8	4.7	6.2	0.9	0.475
β-alanine	21.1	20.6	20.9	1.4	0.964	19	20.7	23.1	2.2	0.387
γ-amino butyric acid	1.7	1.8	1.8	0.1	0.901	0.4	0.6	0.2	0.1	0.070

[1] Values are means ± SE, n = 10. CON = control diet; DL-Met = CON supplement with DL-Met at 25% above the total sulphur amino acids present in the control diet; OH-Met = CON supplement with OH-Met at 25% above the total sulphur amino acids present in the control diet.

Supplementary Figure 1. Allocation of sows and piglets to the three experimental diets (Control, DL-Met and OH-Met) in the gestation, lactation and post-weaning period. CON = control diet; DL-Met = CON supplemented with DL-Met at 25% above the to.

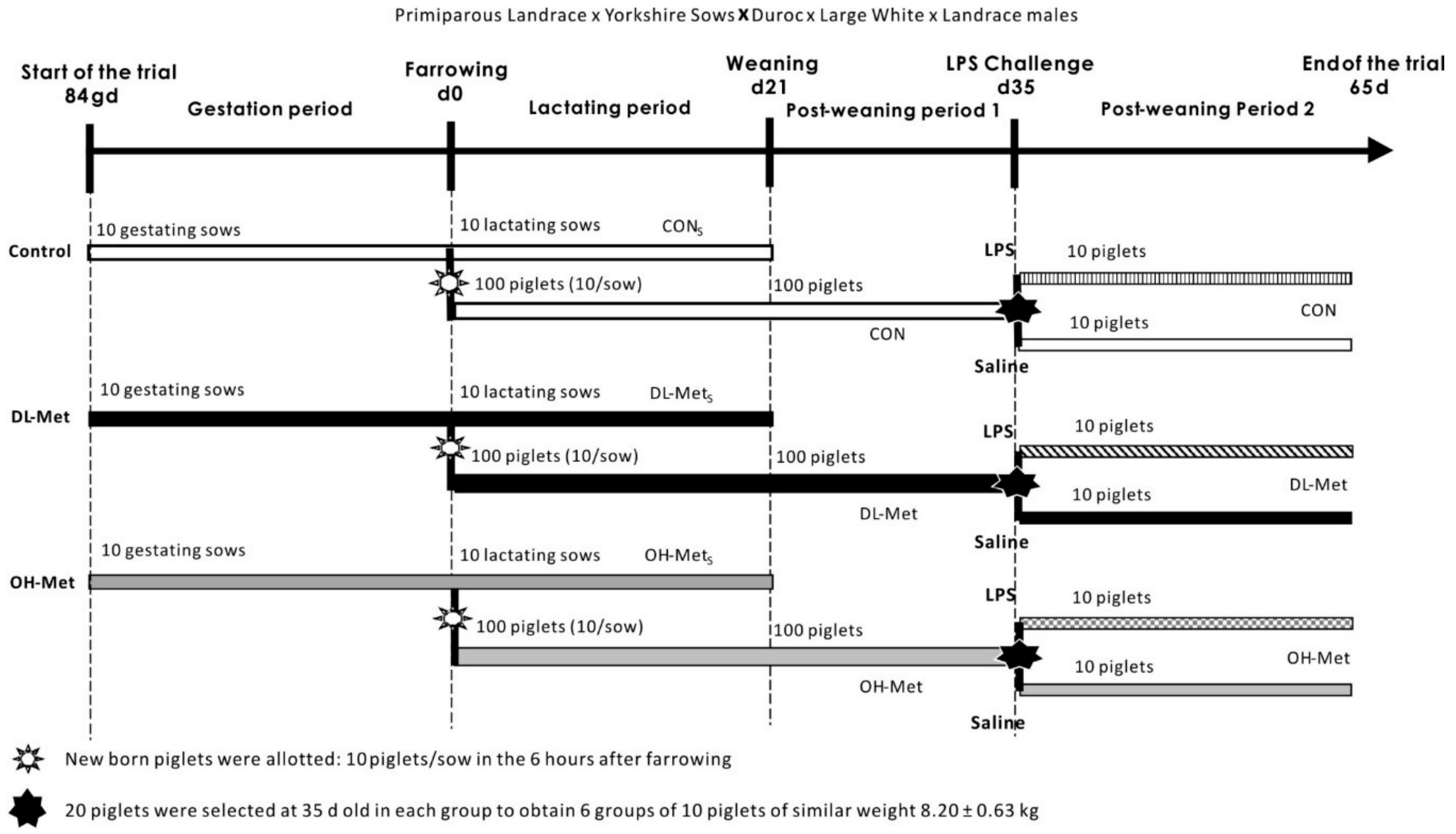

Supplement: Supplementary file 1 [file animals-09-01048-s001.pdf]
